# Discriminant analysis of intermediate brain atrophy rates in longitudinal diagnosis of alzheimer's disease

**DOI:** 10.1186/1746-1596-6-105

**Published:** 2011-10-28

**Authors:** Ali Farzan, Syamsiah Mashohor, Rahman Ramli, Rozi Mahmud

**Affiliations:** 1 Department of Computer & Communication Systems, Faculty of Engineering, University Putra Malaysia, Serdang, 43400, Malaysia; 2 Department of Imaging, Faculty of Radiology, University Putra Malaysia, Serdang, 43400, Malaysia; 3 Institute of Advanced Technology, University Putra Malaysia, Serdang, 43400, Malaysia; 4 Computer Dept., Shabestar branch, Islamic Azad University, Shabestar, Iran

**Keywords:** Alzheimer's disease, diagnostic, discriminate analysis, neuroimaging, whole brain atrophy, principal component analysis

## Abstract

Diagnosing Alzheimer's disease through MRI neuroimaging biomarkers has been used as a complementary marker for traditional clinical markers to improve diagnostic accuracy and also help in developing new pharmacotherapeutic trials. It has been revealed that longitudinal analysis of the whole brain atrophy has the power of discriminating Alzheimer's disease and elderly normal controls. In this work, effect of involving intermediate atrophy rates and impact of using uncorrelated principal components of these features instead of original ones on discriminating normal controls and Alzheimer's disease subjects, is inspected. In fact, linear discriminative analysis of atrophy rates is used to classify subjects into Alzheimer's disease and controls. Leave-one-out cross-validation has been adopted to evaluate the generalization rate of the classifier along with its memorization. Results show that incorporating uncorrelated version of intermediate features leads to the same memorization performance as the original ones but higher generalization rate. As a conclusion, it is revealed that in a longitudinal study, using intermediate MRI scans and transferring them to an uncorrelated feature space can improve diagnostic accuracy.

## 1. Introduction

**Figure 1:**
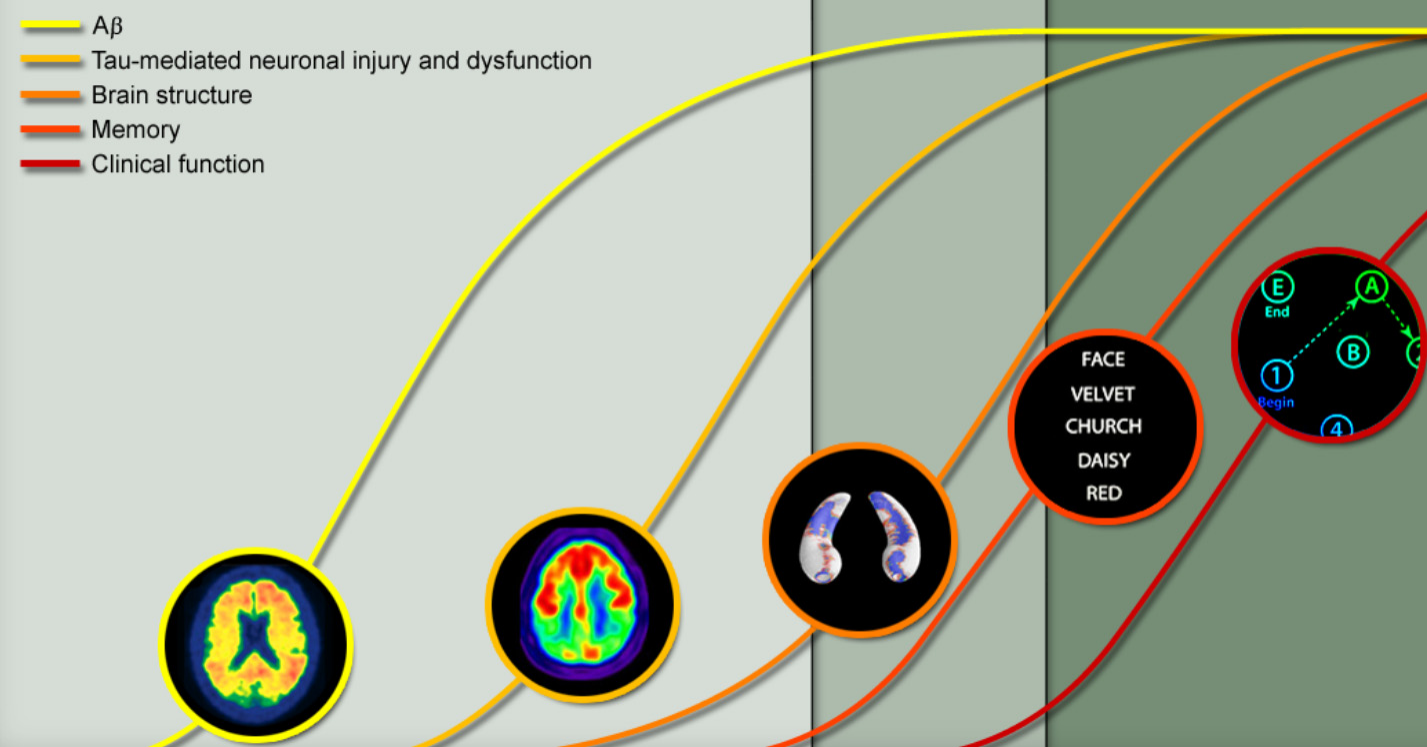
**Various biomarkers of Alzheimer's Disease and the stage of disease they are affective**. The first three biomarkers can be used for prognosis of Alzheimer's Disease prior to dementia diagnosis.

Alzheimer's disease (AD) is known as the most prevalent type of dementia in elderly subjects which has been influenced about 26 million people worldwide [1,2] Disease onset starts with abnormal excessive agglomeration of amyloid β (Aβ) protein and then hyperphosphorylated tau in the brain [1]. This causes deterioration of the synopsis and axons in neurons. Gradually brain degeneration lapses memory and culminates in functional and lingual decline. These changes always intervene in the same order but they may overlap each other in various clinical disease stages [2]. These orders and overlaps are illustrated in Figure 1.

Clinical measures for diagnosing AD are traditionally based on two last biomarker and some standard measures such as Mini Mental Score Exam (MMSE), Clinical Dementia Rating (CDR), Functional Assessment Staging Scale (FAST), Global Deterioration Scale (GDS) or Alzheimer's disease Assessment Scale (ADAS) are used to diagnose people with AD clinically. It is obvious that these measures are useful just in the second and third stages of disease and cannot be used in first stage where there is no manifest behavioral or memory impairment [3,4]. Furthermore, these scores singly are not accurate enough and some complementary biomarkers are needed for accurate diagnosis of AD [4,5]. The need for monitoring disease progression in designing new therapeutic trials encourages researchers to find noninvasive accurate biomarkers of AD [6,7]. MR images due to their high resolution and non-invasive nature, are good candidates for realizing degeneration of brain structures and finding strong relationships between them and disease progression [6]. Various anatomical structures of brain such as Entorhinal Cortex [7-9], Hippocampus [10,11] and Cerebral Cortex [12-14] influenced by AD and their atrophic characteristics such as volume, shape and thickness can be used as biomarkers of AD [6,12,15,16]. Concentrating on atrophic characteristics of anatomical structures is prone to some imperfection. That is, disease related atrophies don't necessarily follow the anatomical boundaries of structures and each part of the brain can be changed under the influence of disease.

**Figure 2:**
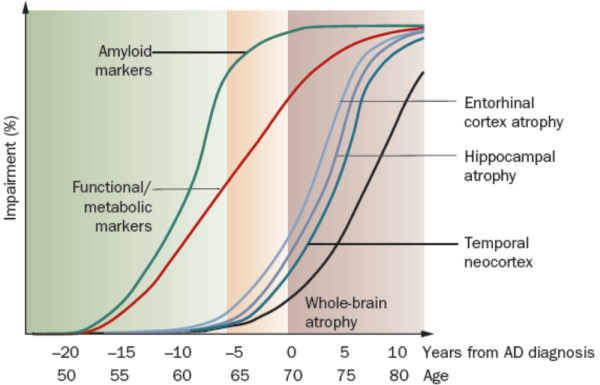
**Natural progression of cognitive and biological markers of Alzheimer disease: a theoretical model**.

The rate of whole brain volume change is almost constant in the third stage of disease and this makes it useful in monitoring the pharmacotherapeutic trials [12,17-19]. Figure 2 shows the profile of structural changes in AD. It is depicted that amyloid markers change at early stages of disease, even decades before diagnosing AD. Besides, degeneration of anatomical structures starts somehow latter, around 10 years before clinically diagnosing AD, but still beneficial for AD prognosis.

There are some methods for measuring brain atrophy in the literature but only three of them are validated. Boundary Shift Integral (BSI) [20,21], Structural Image Evaluation Using Normalization of Atrophy (SIENA) [22] and cross sectional counterpart of it (SIENAX) [18] are the most accurate and broadly accepted methods for evaluating atrophy rate of the brain. Research shows that SIENA has the same accuracy as BSI and so it is fair to choose any of the above-mentioned method in measuring atrophy rate of whole brain in a two-year longitudinal study. That is, the differences between two measures have no effect on the pathological discrimination power of the method.

To measure the whole brain atrophy rate, the pipeline conducted by Smith and et.al are used in this paper [18,23-28]. First step in this pipeline is brain surface extraction which separates the brain from other non-brain parts such as skull or scalp in both images of longitudinal study. To do so, a deformable tessellated mesh have been used which deforms under the control of local parameters and finally matches the brain of head [27]. Afterward, base images must be registered to follow up counterparts. In this step, it was necessary to avoid rescaling artifacts which could change the atrophy size. With this in mind, it has been assumed that the size of skull is constant; it is considered as normalization factor in scaling process. To escape unnecessary modifications of nonlinear registration which matches images as much as possible and eliminates the atrophic differences between them, the linear registration is preferred in this study [26].

Next step is to measure the differences between images. Thus, brain images have been segmented into their three major tissues - Gray Matter (GM), White Matter (WM) and Cerebrospinal Fluid (CSF)- [29]. Boundary points of these tissues have been used to measure the difference between images. One 3 by 3 gradient operator was used to find the gradients in these points. In a peer to peer comparison of 3^mm ^intensity profile on these gradients, the shift distance that maximizes the correlation between these profiles have considered as difference measure. Normalized sum of these measures over all boundary points indicates the overall differences between brain volumes and is called Percentage of Brain Volume Change (PBVC) [22].

Magnetic resonance images (MRI) from Alzheimer's disease neuroimaging (ADNI) database are used in this study [30]. Percentage of brain volume change is evaluated between baseline and the 6th month and the 24th month follow up intervals pair wise. These 3 atrophy rates are used as features in discriminate analysis (DA). Because of high degree of correlation between the features, principal component analysis (PCA) is used to convert the feature space to an uncorrelated feature space and at the same time to reduce the size of space. Discriminative power of these features is compared with the original ones.

## 2. Materials and methods

### 2.1. Subjects

A total of 30 AD patients (46.7% female; mean age of 75 at the standard deviation of 7), and 30 age-matched healthy normal controls (50% female; mean age of 77 at the standard deviation of 5) are selected from the ADNI public database http://www.loni.ucla.edu/ADNI/Data/. ADNI is a large five-year study launched in 2004 by the National Institute on Aging (NIA), the National Institute of Biomedical Imaging and Bioengineering (NIBIB), the Food and Drug Administration (FDA), private pharmaceutical companies and nonprofit organizations, as a $60 million public-private partnership. The primary goal of ADNI has been to test whether serial MRI, PET, other biological markers, and clinical and neuropsychological assessments acquired at multiple sites (as in a typical clinical trial), can replicate results from smaller single site studies measuring the progression of MCI and early AD. Determination of sensitive and definite markers of very early AD progression is destined to aid researchers and clinicians to monitor the effectiveness of new treatments, and diminish the time and cost of clinical trials. The Principal Investigator of this initiative is Michael W. Weiner, M.D., VA Medical Center and University of California, San Francisco.

All the AD and NC subjects in this study had successfully undergone MRI scanning, cognitive tests and clinical evaluation at baseline, 6^th ^months and 2^nd ^year follow up.

### 2.2. Statistical analysis

Some demographic parameters such as age, sex and years of education have remarkable impact on brain atrophic measures and to avoid their influence on the study, subjects of two groups must be matched regarding them. Difference in gender among the two groups is tested with the Chi-square test and matched (*p *= 0.796). Independent two sample student t-test is used to test inter-group differences in age and years of education. As there are no significant differences in age (*p *= 0.188) and years of education (*p *= 0.554) among the two groups, they were ignored in diagnosing AD in this study. Baseline MMSE and PBVC in all three time intervals of baseline to the 6^th ^month follow up (PbvcSc-6), 6^th ^month to 24^th ^month follow up (Pbvc6-24) and baseline to 24^th ^month follow up (PbvcSc-24) indicate significant differences between the two groups (Table 1).

**Table 1:** Demographic and clinical variables by diagnostic group

	NC(*n *= 30)	AD(*n *= 30)	*Ρ*	*Total*
Gender(M/F)	15/15^a^	16/14	0.796	
Age(M/SD)	77/5^a^	75/7	0.188	
Years of Education(M/SD)	16.2/2.9^a^	15.7/2.7	0.554	
Baseline MMSE(M/SD)	29.3/0.8^b^	23.5/2.2	< 0.00001	
PbvcSc-6 (M/SD)	-0.36/0.59^b^	-0.98/0.95	0.005	-0.67/0.87
Pbvc6-24 (M/SD)	-1.24/0.89^b^	-3.11/1.23	< 0.00001	-2.17/1.43
PbvcSc-24 (M/SD)	-1.65/1.05^b^	-4.13/1.85	< 0.00001	-2.88/1.95

Chi-square was used for gender comparison.

Unpaired student t-test was used for age, education-year, MMSE scores and percentage of whole brain volume change (all three) comparisons.

^a ^Indicates insignificant compared to NC group.

^b ^Indicates significant compared to NC group.

These results approve that the two groups are disparate based on longitudinal volume changes, but it does not specify the way of classifying one individual subject into one of these groups based on above features.

DA is a statistical technique used to differentiate groups when the underlying features are quantitative and normally distributed [31]. It is an appropriate method for classifying patterns of subjects into two desired separated groups, AD and NC.

### 2.3. Discriminant analysis

The aim of DA is to analyze group separation power for a set of normally distributed features or pattern of features. Test of normality for all three atrophic measures imply their normal distribution through both groups (Table 2).

**Table 2:** Normality test of atrophy rates using kolmogorov-smirnov method

	NC	AD
PbvcSc-6	0.200*	0.125
Pbvc6-24	0.200*	0.200*
PbvcSc-24	0.200*	0.200*

*. This is a lower bound of the true significance

The simplest and first way to this is using total means of features as threshold values. Patterns with feature values above it will be assigned to one group and the ones bellow it to the other.

Referring to the total means of Table 1, results of classification will be as shown in Table 3. It is obvious that long-term atrophy rates yield higher accuracy.

**Table 3:** Classification based on total mean thresholding

	Threshold Value	Sensitivity	Specificity	Accuracy
PbvcSc-6 (M/SD)	-0.66752	50%	60%	55%
Pbvc6-24 (M/SD)	-2.17367	76.66%	83.33%	80%
PbvcSc-24 (M/SD)	-2.88472	83.33%	93.33%	88.33%

*. Highest accuracy achieved by 24 month longitudinal atrophy rate

**Figure 3:**
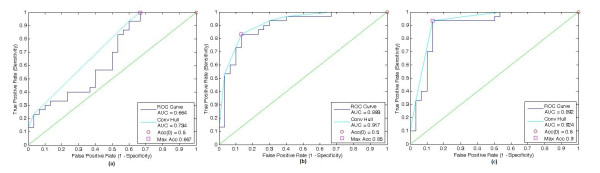
**Receiver Operating Characteristic curve plot for (a) Baseline to 6^th ^month atrophy rate, (b) 6^th ^month to 24^nd ^month atrophy rate, (c) Baseline to 24^nd ^month atrophy rate**. It is conspicuous that using long term atrophy rates for diagnosis, leads to higher accuracy.

These values may not be the optimal threshold values and for comprehensive evaluation, Receiver Operating Characteristic (ROC) curve analysis is carried out. ROC curve plots for all of the three features and associated parameters are shown in Figure 3.

The highest diagnostic accuracy of 90% is achieved by using PbvcSc-24 and a specific threshold value. To evaluate generalization capacity of this feature, leave-one-out-cross-validation is conducted. Finding discloses lower generalization accuracy besides the memorization (Table 4).

**Table 4:** cross validation results

		Predicted	
		NC	AD
Original	NC	90%	10%
	AD	20%	80%

85% of cross-validated cases correctly classified

After that, two other features are included in DA to see whether the accuracy is enhanced or not. A key assumption of DA is that the features should not be highly correlated, but these three features are highly correlated (Table 5).

**Table 5:** correlation coefficients

	PbvcSc-6	Pbvc6-24	PbvcSc-24
PbvcSc-6	1	0.394	0.749
Pbvc6-24	0.394	1	0.899
PbvcSc-24	0.749	0.899	1

*. High correlation between PbvcSc-24 and two other features

It is clear that PbvcSc-24 has high correlation with PbvcSc-6 and Pbvc6-24 and this violates the terms of analysis. To overcome this we use principal component analysis (PCM) to convert them to uncorrelated features. There are two main steps in conducting PCA:

• Step 1: Assessment of data suitability

Sample size or factorability of data, and the strength of the relationship among the features are two main issues to consider in determining whether a particular data set is suitable for PCA or not. A sample size over feature space dimension ratio of 10/1 has been recommended [32]. To put it in other words, at least 10 samples for each feature are needed to be PC analyzed. This criterion is passed in the study. Moreover, two statistical measures are also available for analyzing suitability of the sample size. Bartlett's test of sphericity [33], and the Kaiser-Meyer-Olkin (KMO) measure of sampling adequacy [34]. The Bartlett's test of sphericity should be significant (p < 0.05) and the KMO index which ranges from 0 to 1, should be greater than 0.6 for the PCA to be considered appropriate. These two measures for our dataset are shown in Table 6.

**Table 6:** KMO and Bartlett's Test

KMO Measure of Sampling Adequacy	0.221	0.646
Bartlett's Test of Sphericity	Approx. Chi-Square	292.451
	df	3
	Sig.	< 0.00001

KMO measure is greater than 0.6 and test of sphericity is significant

Factorability of data samples are also confirmed according to these measures. In order for feature relationship to be strong, correlation between features should be at least 0.3 which is at this rate in our case (Table 5).

• Step 2: Feature extraction

In this step the number of features involved in discriminating groups, should be specified. This involves balancing two contradicting needs which are the need to find a simple solution with as few factors as possible and the need to explain as much of the variance in the original data set as possible. There are a number of techniques that can be used to specify the number of features to be kept. One of them is Kaiser's criterion [35], according to which, only features with an eigenvalue of 1.0 or more are retained. The eigenvalue of a feature represents the amount of the total variance explained by that feature. Extracting features by this method leads to selecting only one feature (Table 7).

**Table 7:** Parallel analysis

Component	Total Eigenvalues	Random Eigenvalues
1	2.381	1.1624
2	.615	0.998
3	.004	0.8396

*. Eigenvalues of the real and random generated features

**Figure 4:**
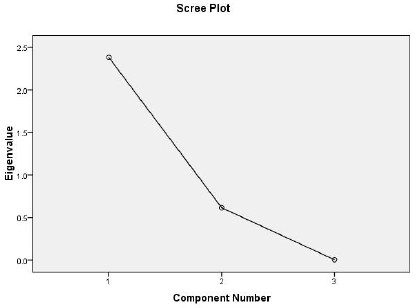
**Breaking happens in feature 2**.

The next test is known as Scree test [36]. It plots each of the eigenvalues and inspects the plot to find a point at which the shape of the curve changes direction toward horizontal or an elbow. Keeping all factors above the elbow is recommended, as these features contribute the most to the explanation of the variance in the data set. In the case of our study, two of the features settle above the elbow and can be kept (Figure 4).

Other method in determining number of features is parallel analysis [37]. Parallel analysis involves comparing the value of the eigenvalues with those obtained from a randomly generated data set of the same size. Only those eigenvalues that exceed the corresponding values from the random data set are kept. According to this analysis, only one of features can be kept (Table 8).

**Table 8:** Total Variance Explained

	Initial Eigenvalues			Extraction Sums of Squared Loadings		
Component	Total	% of Variance	Cumulative %	Total	% of Variance	Cumulative %
1	2.381	79.371	79.371	2.381	79.371	79.371
2	.615	20.492	99.863			
3	.004	.137	100.000			

Extraction Method: Principal Component Analysis

Regarding to the three abovementioned methods, only one of the features must be selected for discriminating subjects. Referring to the Table 7, it carries 79.371% of total variance among data which seems not satisfactory. Indeed, PCA is used as a data exploration technique, so the interpretation and the way we use it is up to our judgment, rather than any hard and fast statistical rules. Here in this article, it is supposed that the algorithm is interested only in components that have an eigenvalue of 0.6 or more. By extracting two uncorrelated features, with which 99.863% of total variance among data will be carried, which is highly satisfactory.

To investigate the contribution degree of initial features in newly extracted ones, refer to Table 9. It can be seen from this table that most of the features load quite strongly (above 0.4) on them (except PbvcSc-6 on PC2).

**Table 9:** component matrix

Features	Extracted feature 1 (PC1)	Extracted feature 2 (PC2)
PbvcSc-24	0.997	0.613
Pbvc6-24	0.874	-0.485
PbvcSc-6	0.789	-0.061

Pattern of loading for extracted features

As expected, the new extracted features are highly uncorrelated (Table 10).

**Table 10:** within group CORRELATION MATRIX

Features	PC1	PC2
PC1	1	-0.099
PC2	- 0.099	1

Extracted features are highly correlated

DA can be carried on by these two newly extracted uncorrelated features.

Calculated unstandardized canonical discriminant function is:

(1) ds=(0.347*PC1)-(0.592*PC2)+2.062

With *ds *as discriminant score, Table 11 shows the mean of *ds *for two groups of subjects which are conspicuously far apart each other.

**Table 11:** discriminant function at group Centroid

Group	Mean *ds*
NC	0.89
AD	- 0.89

Unstandardized canonical discriminant functions evaluated at group means

To measures the association between the *ds *and the groups, Canonical correlation should be considered (Table 12). A high value (near 1) shows that the function discriminates quite well.

**Table 12:** Eigenvalues

Function	Eigenvalue	% of Variance	Cumulative %	Canonical Correlation
1	.820^a^	100.0	100.0	.671

Canonical discriminant function were used in the analysis

With regard to canonical correlation of 0.671 in this study, discrimination power of these extracted features is conceived as moderate. Wilk's Lambda shows the proportion of the total variance (55%) in the *ds *not explained by differences among groups (Table 13). A small Lambda value (near 0) indicates that the group's mean *ds *differs. The Sig (p < 0.001) is for the Chi-square test which indicates there is a highly significant difference between the groups' centroids.

**Table 13:** Wilks' Lambda

Test of Function(s)	Wilks' Lambda	Chi-square	df	**Sig**.
1	0.55	34.124	2	< 0.00001

Centroids of groups are significantly different

To investigate the impact of each extracted feature on the discriminant function, correlation (in order of importance) of each feature with the *ds *is calculated (Table 14). It is revealed that PC1 has highest impact on discrimination process.

**Table 14:** Structure Matrix

Group	Mean *ds*
PC1	0.927
PC2	- 0.466

First extracted feature has highest correlation with *ds*

## 3. Results and discussion

As the final stage in DA, the classification results are summarized in Table 15.

**Table 15:** classification results

		Predicted	
		NC	AD
Original	NC	93.3%	6.7%
	AD	16.7	83.3%

88.33% of original cases correctly classified

Results show that there is not any improvement in the accuracy of the model with two extracted features (PC1-PC2) compared to PBVCsc24 alone (88.33%). To indicate that the discriminatory power of the classification is statistically better than done by chance (50%), Press's Q statistic is used to compare with the critical value (6.63) from the Chi-square distribution.

(2) $$\text{press’s}\;\text{Q}\;\text{statistic}=\frac{{\left[N-nk\right]}^{2}}{N\left(k-1\right)}$$

where *N *is total sample size, *n *is the number of correctly classified patterns and *k *is the number of different groups. It is evaluated to 35.27 which is greater than the critical value of 6.63:

$$\frac{{\left[60-53\;*\;2\right]}^{2}}{60\left(2-1\right)}=\frac{46^{2}}{60}=35.27$$

So, the results of achieved classifier are better than classified by chance. To evaluate the generalization capacity of this classifier, we involved leave-one-out cross validation method. Results are shown in Table 16.

**Table 16:** cross validation results

		Predicted	
		NC	AD
Original	NC	93.3%	6.7%
	AD	16.7%	83.3%

88.3% of cross-validated cases correctly classified

Compared to the generalization results of initially selected features in Table 4, it can be seen that the accuracy of the diagnosis using two extracted uncorrelated features (PC1-PC2) improves, compared to PBVCsc24 alone for about 3.33%. It is revealed in Table. 17.

## 4. Conclusion

Findings of the study disclose that in longitudinal analysis of brain atrophy rate for diagnosing AD subjects, incorporating some intermediate (between baseline and follow up) MRI scans and using their corresponding atrophy rates in uncorrelated form or principal components of them, can improve the accuracy of diagnosis specially from generalization aspect.

In spite of this improvement, linear classifiers cannot discriminate subjects with the highest accuracy expected in the ROC curve. Consequently, nonlinear classifiers such as kernel support vector machine (SVM) must be invoked to achieve a higher accuracy of diagnosis. This is mainly because of nonlinear nature of atrophy rate between the subjects.

## Appendix

### Cross validation

In ***k***-fold cross-validation, the initial data set is randomly partitioned into *k *non-overlapping subsets or "folds" (*D*_1_, *D*_2_, ... , *D_k) _*each of which with approximately equal size. Training and testing is performed *k *times. In iteration *i*, subset *D_i _*is reserved as test set, and the remaining subsets are collectively used to train the model. To put it simple, in the first iteration, subsets *D*_2_, ... , *D_k _*are used as the training set in order to obtain a first model, which is tested on *D*1; the second iteration is trained on subsets *D*_1_, *D*_3_, ..., *D_k _*and tested on *D*_2_, and so on. For classification, the accuracy estimation is the overall number of correct classifications from the *k *iterations, divided by the total number of tuples in the initial data.

Leave-one-out is a special case of *k*-fold cross-validation where *k *is set to the number of initial tuples. That is, only one sample is left out at a time for the test set.

### Principal Component Analysis (PCA)

It is a way of identifying patterns in data, and expressing the data in such a way as to highlight their similarities and differences [38]. The other main advantage of PCA is that once you have found these patterns in the data, you can compress the data by reducing the number of dimension, without much loss of information. This technique is used in feature extraction to reduce feature space dimension and make features more discriminative.

PCA involves the eigenvalue decomposition of data covariance matrix to generate features that are optimally uncorrelated

$$I\left(i_{1},\;\;i_{2},\;\;i_{3},\cdots \;,\;i_{m}\right)=A^{T}\cdot P\left(p_{1},\;p_{2},\;p_{3},\;\cdots \;,p_{n}\right)$$

Where *P *is the original pattern of features and *I *is the pattern of uncorrelated features. *A *is the eigenvalue of covariance matrix.
